# Potential Piperolactam A Isolated From *Piper betle* as Natural Inhibitors of *Brucella* Species Aminoacyl‐tRNA Synthetase for Livestock Infections: In Silico Approach

**DOI:** 10.1002/vms3.70042

**Published:** 2024-09-24

**Authors:** Diding Latipudin, Sefren Geiner Tumilaar, Yoga Ramdani, Dudi Dudi, Dikdik Kurnia

**Affiliations:** ^1^ Department of Animal Nutrition Faculty of Animal Husbandry Universitas Padjadjaran Sumedang West Java Indonesia; ^2^ Department of Chemistry Faculty of Mathematics and Natural Sciences Universitas Padjadjaran Sumedang West Java Indonesia

**Keywords:** aminoacyl‐tRNA synthetases (aaRS), antibacterial, *Brucella* sp, in silico, *P. betle*, piperolactam A

## Abstract

Brucellosis is an important global zoonosis caused by the bacterium *Brucella* sp. Brucellosis causes abortions, reproductive failure and reduced milk production, resulting in significant economic losses. *Brucella* species are reported to be resistant to antibiotics, which makes treatment difficult. The urgency of discovering new drug candidates to combat *Brucella*’s infection necessitates the exploration of novel alternative agents with unique protein targets. Aminoacyl‐tRNA synthetases (aaRSs), which have fundamental functions in translation, inhibit this process, stop protein synthesis and ultimately inhibit bacterial growth. The purpose of this study was to isolate piperolactam A compounds from the methanol extract of *Piper betle* leaves that have potential as antibacterials to inhibit the growth of *Brucella* sp. causing brucellosis in livestock and to analyse the mechanism of inhibitory activity of piperolactam A compounds against the aaRS enzyme through a molecular docking approach in silico. Piperolactam A was isolated from *P. betle* by column chromatography and characterized by UV, IR, 1D and 2D NMRs and MS, then tested for their inhibition mechanism against the enzymes threonyl‐tRNA synthetase, leucyl‐tRNA synthetase (LeuRS) and methionyl‐tRNA synthetase in silico. The result in silico test is that piperolactam A has the potential to inhibit LeuRS enzyme with the greater binding affinity.

## Graphical abstract

Piperolactam A (**1**) was successfully isolated from *P. betle* plant, which is strongly predicted to inhibit the activity of aminoacyl‐tRNA synthetases, such as ThrRS, LeuRS and MetRS receptor enzymes in silico. Piperolactam A (**1**) acts on the active site tethering of the third amino acid residue of this receptor and has a fairly strong binding affinity.

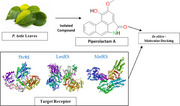

## 1 Introduction

Brucellosis is an important global zoonosis caused by the bacterium *Brucella* sp. (Gwida et al. 2010; Holt et al. 2021; Li, Wen, and Zhao 2016). Zoonoses are transmitted from animals to humans through contaminated food, including dairy products. They can also be transmitted by direct contact with infected animals or by inhalation of aerosols (Ojo et al. 2016). The zoonotic potential of *Brucella abortus* (cattle), *Brucella melitensis* (sheep and goats) and *Brucella suis* (pigs and swine) is considered high, as they are responsible for most human infections caused by *Brucella* (Golshani et al. 2016; von Bargen, Gorvel, and Salcedo 2012). In many developing countries, brucellosis represents a major challenge to the global livestock industry and a threat to human health. Annually, globally, there are more than 500,000 human infections of brucellosis (Gwida et al. 2010; Lopes, Nicolino, and Haddad 2010; Rowaiye, Ogugua, and Ibeanu 2022). The disease is geographically limited. However, it remains a major public health challenge in the Mediterranean, Africa, Asia and Latin America. Brucellosis causes abortions, reproductive failure and reduced milk production, resulting in significant economic losses (Wareth, Dadar, and Ali 2022; Zamri‐Saad and Kamarudin 2016).

*Brucella* species are reported to be resistant to antibiotics, which makes treatment difficult. These bacteria could reside inside the host's cells and are able to evade the immune response and inhibit programmed cell death, which provides them with an extended life span (Kumari et al. 2017; Trott, Abraham, and Adler 2018). Natural products remain an important source for the discovery of new antibacterial drugs and have been used as effective therapeutics against bacterial infections (Dai et al. 2022). Antimicrobial drugs can still be used on livestock in order to encourage the development of healthier and more productive animals (Page and Gautier 2012). The urgency of discovering new drug candidates to combat *Brucella*’s infection necessitates the exploration of novel alternative agents with unique protein targets.

Aminoacyl‐tRNA synthetases (aaRSs), which have fundamental functions in translation, catalyse the covalent linkage of amino acids to their corresponding tRNAs, creating aminoacyl tRNAs (aa‐tRNA) for protein synthesis (Kwon, Fox, and Kim 2019; Rajendran et al. 2018). Therefore, inhibiting this process stops protein synthesis and ultimately inhibits bacterial growth (Hurdle, O'Neill, and Chopra 2005). The tRNA synthetases can be classified into two types on the basis of the mutually exclusive sequence motifs that can express different active site topologies. The first class includes several aaRSs, such as arginyl tRNA synthetase (ArgRS), methionyl‐tRNA synthetase (MetRS) and leucyl‐tRNA synthetase (LeuRS), all of which contain a classical Rossmann dinucleotide–binding domain. The second class includes mainly threonyl‐tRNA synthetase (ThrRS), histidyl tRNA synthetase (HisRS), glycyl tRNA synthetase (GlyRS) and others. These enzymes have new anti‐parallel β‐sheets (Pang, Weeks, and Van Aerschot 2021; Travin, Severinov, and Dubiley 2021; Zhang and Ma 2019).

In addition, there are significant structural differences between eukaryotic and prokaryotic aaRSs. These differences provide a structural basis for the development of selective inhibitors of bacterial aaRSs. Given their essential role in translation, aaRS are considered highly promising targets for developing antibiotics in pathogenic species (Kim, Bae, and Song 2020; Zhang and Ma 2019). Developing new inhibitors of aaRS, especially ThrRS, LeuRS and MetRS, as antimicrobial drugs have great potential and importance for developing new antimicrobials (Zhang and Ma 2019). The main reason for selecting these three receptors is based on their biological relevance, including LeuRS is crucial for protein synthesis as it attaches leucine to its corresponding tRNA. This enzyme is involved in a wide range of cellular processes, including the regulation of protein synthesis and cell signalling. Its dysfunction can lead to various diseases, making it an important target for drug discovery (Wang et al. 2014). MetRS initiates protein synthesis by attaching methionine to tRNA. Because methionine is the first amino acid incorporated into nascent proteins, MetRS plays a critical role in the initiation phase of translation. It is also a target for antibiotics and anti‐cancer drugs, making it a significant enzyme for pharmacological studies (Kim and Jung 2024). ThrRS is responsible for attaching threonine to its corresponding tRNA. It is involved in protein synthesis and has unique structural features that can be exploited for specific drug design. Its role in cellular function and disease contexts justifies its inclusion in studies aiming to discover novel inhibitors or modulators (Ruan et al. 2015).

This study focused on isolating and characterizing piperolactam A from *Piper betle*. The primary aim was to evaluate the potential of this compound using in silico docking studies to predict its activity against specific aaRS. Thus, the study primarily involved predicting the interactions of piperolactam A with targeted aaRS (ThrRS, LeuRS and MetRS) using computational methods.

## 2 Materials and Methods

### 2.1 Materials and Chemicals for Isolation

The main materials used for isolation were betel leaf extracts cultivated in Bandung City, West Java Province, Indonesia, from 2019 by local farmers. The leaves were identified and registered at the Department of Biology, Laboratory of Taxonomy, Universitas Padjadjaran, Indonesia. The chemicals were organic solvents, namely methanol, *n*‐hexane, ethyl acetate and chloroform, then analytical organic solvents from Merck Co. Ltd., Darmstadt, Germany and Sigma Aldrich Co. Ltd. (St. Louis, MO, USA) for the spectroscopic analysis. The column chromatography materials were Silica G 60 (Merck) and ODS RP‐18. Then, the thin‐layer chromatography (TLC) for the spot compounds was Silica G 60 F_254_ and ODS RP‐18 F_254_S (Merck).

### 2.2 Instruments

The instruments used for characterization were spectrophotometer UV (8452A Dipde Array, Hewlett Packard Palo Alto, CA, USA), FTIR Shimadzu 8400 (SpectraLab Scientific Inc., Markham, Canada), 1D and 2D‐NMR by JEOL type ECA 500 MHz (JEOL Ltd., Tokyo, Japan) and mass spectrometry (Water Acquit UPLC type triquadrupole, Agilent, CA, USA).

### 2.3 Isolation and Characterization of Piperolactam A From *P. betle*

The isolation of piperolactam A from *P. betle* was carried out using column chromatography. *P. betle*–dried leaves (10 kg) were extracted using methanol (100 L) by maceration for 3 days and concentrated by rotary evaporator to obtain the crude solid extract with a yield of 52.3 g. The stationary phase used is Silica G 60 with a gradient solvent system, and the mobile phase is starting from *n*‐hexane (100%) to ethyl acetate (100%). The methanol extract (30 g) was put into a column that already contained Silica G 60 and then isolated with a gradient of 10% to obtain eleven fractions (F1–F11). The eighth fraction (335 mg) was purified with a gradient solvent system from water (100%) to methanol (100%) by increasing the methanol by 10.5% to obtain 21 subfractions (F8.1–F8.21). After that, F.8.11–12 (39 mg) was purified again with a gradient solvent system from *n*‐hexane (100%) to chloroform (100%) by increasing the chloroform by 10.5% and 2%. The stain pattern of this compound was monitored using TLC Silica G 60 F_254_ and TLC ODS RP‐18. By TLC profile, subfraction F.8.(11–12).15 showed a single spot as compound 1 (1.6 mg). Then, the characterization of Compound 1 was characterized using ^1^H‐NMR, ^13^C‐NMR, HMQC, HMBC and COSY by JEOL JNM‐ECA500 MHz (JEOL Ltd.) with CD_3_OD as the solvent. The mass spectrum was obtained using a water‐acquitted UPLC‐type triquadrupole Agilent MS system with electrospray ionization (ESI) in positive mode. The IR spectrum was recorded on a PerkinElmer Spectrum 8400 FTIR spectrometer using KBr pellets.

### 2.4 Molecular Docking Against aaRS Enzyme

The structures of aaRS enzyme were extracted from the RCSB protein data bank (https://www.rcsb.org) with PDB IDs of ThrRS 1QF6, LeuRS 4AQ7 and MetRS 4DLP. The compound piperolactam A was obtained from the PubChem compound database (https://pubchem.ncbi.nlm.nih.gov/), with the CID of piperolactam A being CID‐3081016. The compound was obtained from PubChem compound database with the canonical SMILE of piperolactam A COC1 = C(C2 = C3C(=C1)C(=O)NC3 = CC4 = CC = CC = C42)O. The structures were converted to three‐dimensional structures using ChemDraw 3D format. The aaRS enzymes were obtained from the RCSB protein database, with PDB IDs of ThrRS is 1QF6, LeuRS is 4AQ7 and MetRS is 4DLP, respectively. The proteins were prepared by removing water molecules, adding hydrogen atoms and assigning appropriate charges using AutoDockTools. The native ligand was isolated from the enzyme macromolecule by the BIOVIA Discovery Studio programme. The docking studies were conducted using AutoDock4. The binding site was defined on the basis of the active site of the protein or known ligand‐binding site. The grid box was set to cover the binding site adequately. The grid box area for the 1QF6 protein was set with the following parameters: Grid box area *x* = 45.0 Å, *y* = 50.0 Å, *z* = 55.0 Å, whereas grid box area for 4AQ7 protein was set with *x* = 25.0 Å, *y* = 30.0 Å, *z* = 35.0 Å, and grid box area for 4DLP was set with *x* = 40.0 Å, *y* = 45.0 Å, *z* = 50.0 Å. The docking results were analysed on the basis of the binding affinity and interaction profiles. The molecular interaction was visualized by using BIOVIA discovery studio programme.

## 3 Results

### 3.1 Isolation and Characterization of Piperolactam A From *P. betle*

Piperolactam A (1.6 mg) has been isolated from *P. betle* with yellow powder. It can fluoresce at UV 254, UV 356 and H_2_SO_4_. The FTIR spectrum of piperolactam A was used to predict the functional group of the compound. Piperolactam A has NH group (3478 cm^−1^), OH group (3185 cm^−1^), C–H sp^3^ (2925 cm^−1^), C = O (1655 cm^−1^), C–N (1384 cm^−1^) and C–O (1130 cm^−1^).

Structure elucidation of piperolactam A was carried out using 1D and 2D NMR spectra. The first step is to analyse ^13^C‐NMR and DEPT 135° spectra to determine the amount and type of carbon. These carbons have a chemical shift of 57.4 ppm (CH_3_ sp^3^), 107.0 ppm (CH sp^2^), 108.6 ppm (CH sp^2^), 115.8 ppm (C quartener), 116.6 ppm (C quartener), 125.9 ppm (C quartener), 126.2 ppm (CH sp^2^), 127.5 ppm (CH sp^2^), 128.5 ppm (C quartener), 128.9 ppm (CH sp^2^), 129.6 ppm (CH sp^2^), 135.5 ppm (C quartener), 135.6 ppm (C quartener), 149.5 ppm (C quartener), 151.4 ppm (C quartener) and dan 172.1 ppm (C quartener). Therefore, one methyl, six methines and nine quaternary carbons are obtained.

Analysis of spectrum HMQC was used to determine the ownership of protons to carbon. Protons 4.07 ppm is correlated to C1, protons 7.14 ppm is correlated to C2, protons 7.75 ppm is correlated to C3, protons 7.50 ppm is correlated to C7 and C8, protons 9.40 ppm is correlated to C10 and protons 7.82 ppm is correlated to C11. Then, this correlation is used for the number of protons in the ^1^H‐NMR spectrum.

The ^1^H‐NMR spectrum was used to determine the number, type, proton environment and partial structure of the compound. Table 1 shows the chemical shift of the protons, namely 4.07 ppm (3*H*, s), 7.14 ppm (1*H*, s), 7.50 ppm (2*H*, m), 7.75 ppm (1*H*, s), 7.82 ppm (1*H*, m) and 9.40 ppm (1*H*, m).

**TABLE 1:** Data of ^1^H‐NMR and ^13^C‐NMR spectra of piperolactam A.

Atom	Experiment		Atom	Lin et al. (2013)		Salleh et al. (2019)	
	*δ*_C_ (ppm)	*δ*_H_ (ppm)		*δ*_C_ (ppm)	*δ*_H_ (ppm)	*δ*_C_ (ppm)	*δ*_H_ (ppm)
C‐1	57.4	4.07 (3*H*, s)	–OCH_3_	57.5	4.10 (3*H*, s)	57.7	4.09 (3*H*; s)
C‐2	107.0	7.14 (1*H*, s)	C‐9	107.0	7.15 (1*H*, s)	107.2	7.15 (1*H*, s)
C‐3	108.6	7.75 (1*H*, s)	C‐2	108.5	7.75 (1*H*, s)	108.9	7.77 (1*H*, s)
C‐4	115.8	—	C‐4a	116.1	—	116.0	—
C‐5	116.6	—	C‐1	116.5	—	116.9	—
C‐6	125.9	—	C‐10a	126.5	—	126.2	—
C‐7	126.2	7.50 (1*H*, m)	C‐6	126.5	7.55 (1*H*, m)	126.4	7.53 (1*H*, m)
C‐8	127.5	7.50 (1*H*, m)	C‐7	127.5	7.25 (1*H*, m)	127.7	7.53 (1*H*, m)
C‐9	128.5	—	C‐4b	128.5	—	128.8	—
C‐10	128.9	9.30 (1*H*, m)	C‐5	129.0	9.30 (1*H*, m)	129.2	9.32 (1*H*, m)
C‐11	129.6	7.82 (1*H*, m)	C‐8	129.5	7.85 (1*H*, m)	129.8	7.85 (1*H*, m)
C‐12	135.5	—	C‐8a	135.8	—	135.7	—
C‐13	135.6	—	C‐10	135.8	—	135.9	—
C‐14	149.5	—	C‐3	149.7	—	149.7	—
C‐15	151.4	—	C‐4	151.5	—	151.6	—
C‐16	172.1	—	C = O	172.2	—	172.3	—

On the basis of FTIR, ^13^C‐NMR, DEPT 135°, HMQC and ^1^H‐NMR spectra, it can be determined that the structure formula is C_16_H_11_NO_3_ with a double bond equivalent (DBE) of 12. This value indicates conjugated benzene ring (phenanthrene) and γ‐lactam in the compound. Furthermore, the analysis of HMBC and ^1^H‐^1^H COSY spectra was carried out to strengthen the alleged compound. HMBC spectrum was used to determine the correlation of proton and carbon with a distance of three to four bonds. The spectrum indicates H1 correlated to C14, H2 to C6 and C9, H3 to C6, C15 and C16, H10 to C8 and C12 and H11 to C7 and C9. Then, the analysis of the ^1^H‐^1^H COSY spectrum is to determine the correlation of proton and proton with the distance of three bonds. The spectrum indicates H7 correlated to H10 and H8 correlated to H11.

On the basis of those spectra, it can be confirmed that piperolactam A has been isolated from *P. betle*. Table 1 is the comparison of proton and carbon chemical shifts between the experiment and the previous study (Lin et al. 2013; Salleh et al. 2019). The peaks of these three data are not significantly different. Therefore, it can be confirmed that the isolated compound is piperolactam A. This structure was also confirmed by mass spectrum analysis. The spectrum indicates the value of [M − H]^+^ 266.08, which corresponds to the molecular weight of piperolactam A 265 g/mol.

### 3.2 Molecular Docking Against aaRSs

A prediction mechanism was carried out between piperolactam A and antibiotics to ThrRS, LeuRS and MetRS enzymes using AutoDock 4.0 tools. Table 2 shows the binding affinities and inhibition constants of piperolactam A, borrelidin and purpuromycin to ThrRS. Borrelidin was used for positive control of ThrRS, and purpuromycin was used for positive control of multiple aaRSs.

**TABLE 2:** Prediction of binding affinity and inhibition constant of compounds against threonyl‐tRNA synthetase (ThrRS) enzyme.

No.	Compounds	Binding affinity (kcal/mol)	Inhibition constant/Ki (µM)
1	AMP (native)	−7.82	1.86
2	Piperolactam A	−7.35	4.11
3	Borrelidin	−7.77	2.01
4	Purpuromycin	−7.61	2.64

Furthermore, BIOVIA Discovery Studio programme was used for the visualization of the interaction between amino acid residues of the enzymes to piperolactam A and antibiotics. Table 3 shows several amino acids which interact with piperolactam A, borrelidin and purpuromycin by hydrogen bond and hydrophobic and electrostatic interactions.

**TABLE 3:** Molecular interactions to residues of threonyl‐tRNA synthetase (ThrRS) enzyme.

Category	Type of interaction	AMP (native)	Piperolactam A	Borrelidin	Purpuromycin
Hydrogen bond	Conventional	Glu365, Val376, Gln479, Cys480, Ser517	Glu365, Arg375	Tyr313, Tyr462, Thr482	Arg363, Glu365, Val376, Gly459, Arg520
	Carbon	Gln381, Cys516, Gly516	Leu373	—	Leu373, Met374, Arg375, Glu458, Ala460, Ser517
Hydrophobic	Pi–alkyl	Phe379	Phe379	Met332, Ala460, Ala513	Lys45
	Pi–cation	Arg363, Arg520	Arg520	—	Arg363
	Pi–Pi stacked	—	—	—	Phe379
Electrostatic	Van der Waals	Met374, Thr482, Gln484	Arg363, Met374, Gln479, Cys480, Gly516, Ser517, Glu519	His309, Asn333, Cys334, Arg363, Gln381, Asp383, His385, Phe461, Lys465, Gln479, Cys480, Gln484, His511, Gly516, Ser517	Tyr313, Pro424, Gly457, Phe461, Lys465, Glu519

Table 4 shows the binding affinities and inhibition constants of piperolactam A, borrelidin and purpuromycin to LeuRS. Tavaborole was used for positive control of LeuRS, and purpuromycin was used for positive control of multiple aaRSs.

**TABLE 4:** Prediction of binding affinity and inhibition constant of compounds against leucyl‐tRNA synthetase (LeuRS) enzyme.

No.	Compounds	Binding affinity (kcal/mol)	Inhibition constant/Ki (µM)
1	LMS (native)	−8.62	0.48
2	Piperolactam A	−7.47	3.34
3	Tavaborole	−4.53	474.45
4	Purpuromycin	−7.66	2.55

Furthermore, the BIOVIA Discovery Studio programme was used for visualization of the interaction between amino acid residues of the enzymes to piperolactam A and antibiotics. Table 5 shows several amino acids which interact with piperolactam A, tavaborole and purpuromycin by hydrogen bond and hydrophobic and electrostatic interactions.

**TABLE 5:** Molecular interactions to residues of leucyl‐tRNA synthetase (LeuRS) enzyme.

Category	Type of interaction	LMS (native)	Piperolactam A	Tavaborole	Purpuromycin
Hydrogen bond	Conventional	Leu41, Tyr43, His52, Asn55, Gly530, Glu532, Gln566, Val569, Met620	Tyr43, Asn55	Val569	His52, Asp80, Ser496, Glu532, Gln566
	Carbon	His49, Gly567	Met40	His52, Gln566	Gly530, Val569
Hydrophobic	Pi–alkyl	Met568	Met568, Lys619	Val569	Met568
	Pi–sigma	—	—	—	Phe493
	Pi–cation	—	His49, Glu532	—	His533
	Pi–anion	Glu532	His52, Glu532	Glu532, Met568	Glu532
Electrostatic	Van der Waals	Met40, Pro42, Gly51, Arg54, Tyr56, Asp80, Gly529, His533, Lys619	Pro42, Gly51, Tyr56, Gly529, Gln566, Val569, Met620, Ser621	His49, Gly51, Asn55, Gly567, Leu570, Lys619, Met620	Leu41, Pro42, Tyr43, His49, Gly51, Arg54, Asn55, Thr492, Gly529, Gly567, Lys619, Met620

Table 6 shows the binding affinities and inhibition constants of piperolactam A, borrelidin and purpuromycin to MetRS. REP8839 was used for positive control of MetRS, and purpuromycin was used for positive control of multiple aaRSs.

**TABLE 6:** Prediction of binding affinity and inhibition constant of compounds against methionyl‐tRNA synthetase (MetRS) enzyme.

No.	Compounds	Binding affinity (kcal/mol)	Inhibition constant/Ki (µM)
1	MSE (native)	−5.59	80.32
2	Piperolactam A	−6.88	9.03
3	REP8839	−8.92	0.29
4	Purpuromycin	−9.46	0.12

Furthermore, BIOVIA Discovery Studio programme was used for visualization of the interaction between amino acid residues of the enzymes to piperolactam A and antibiotics. Table 7 shows several amino acids which interact with piperolactam A, REP8839 and purpuromycin by hydrogen bond and hydrophobic and electrostatic interactions.

**TABLE 7:** Molecular interactions to residues of methionyl‐tRNA synthetase (MetRS) enzyme.

Category	Type of interaction	MSE (native)	Piperolactam A	REP8839	Purpuromycin
Hydrogen bond	Conventional	Ile12, Asp51	Asp264	Glu26	Tyr14, His23, Tyr135, Asp264
	Carbon	—	Ala11, His23, Glu26, His291	—	Tyr14
Hydrophobic	Pi–alkyl	Ile12, Trp230, Ala233, Leu234, Tyr237, His269	Ala11, Ala13, Ile265	Ala13, His23, Leu27, His291, Phe293	Tyr14
	Pi–sigma	—	Ile265	—	—
	Pi–cation	—	His23	—	Lys56
	Pi–anion	—	Glu26	Asp264	Asp131
	Pi–Pi T‐shaped	—	—	His23	Phe268
	Pi–lone pair	—	—	Thr48	
Electrostatic	Van der Waals	—	Thr10, Ile12, Tyr14, Gly22, Trp230, Gly262, Phe293	Thr10, Ala11, Ile12, Gly22, Gly49, Thr50, Phe79, Met82, Ala83, Tyr93, Ile261, Gly262, Ile265	Ala13, Asn16, Gly17, His20, Ala133, Pro151, Gln152, Tyr228, Val229, Trp230, Ile265

## 4 Discussion

Piperolactam is one of the secondary metabolite compounds, which is a group of alkaloid. Piperolactam A has never been isolated from *P. betle* leaves, and in this study, it was successfully carried out. Until now, the compounds that have been successfully isolated and characterized from *P. betle* leaves are pipercerebroside A, pipercerebroside B (Chen et al. 2013), 1‐*n*‐dodecanyloxy resorcinol, desmethylenesqualenyl deoxy‐cepharadione‐A (Atiya, Sinha, and Ranjan Lal 2018), 1‐*n*‐decanoyl hydroxybenzoic acid, 1‐*n*‐decanoyl phenol and 3‐butylphenol (Atiya et al. 2020). However, piperolactam A has only been isolated from *P. betle* root extract (Amin et al. 2017). This is evidence that this compound has been isolated for the first time from *P. betle* leaves or to support the novelty of isolating piperolactam A. It has several antimicrobial activities; piperolactam A showed specific activity against *B. subtilis* and *Staphylococcus aureus* (Lertnitikul et al. 2023).

On the basis of current literature and available data up to August 2024, piperolactam A has not been reported to have growth inhibition activity specifically against *Brucella* sp. Piperolactam A is known for its various biological activities (Woon, Ahmad, and Zamakshshari 2023), including potential anti‐inflammatory and anti‐cancer effects, but its activity against *Brucella* sp. appears to be unreported in the scientific literature.

Furthermore, a study of molecular docking was also carried out to know the inhibition mechanism. Molecular docking was used to investigate the antibacterial activity of piperolactam A by inhibiting the activity of ThrRS, LeuRS and MetRS enzymes. This means that RNA translation for protein synthesis stops and inhibits bacterial growth (Travin, Severinov, and Dubiley 2021). ThrRS is an aaRS that specifically transfers l‐threonine to tRNA^Thr^, forming threonyl‐tRNA, which decodes the l‐threonine codons on mRNAs during protein translation (Guo et al. 2020). Borrelidin is a non‐competitive inhibitor of ThrRS and exhibits a wide spectrum of biological activities (Li et al. 2014). The leuS gene encodes LeuRS, which catalyses the aminoacylation of tRNA^Leu^ by reacting with three substrates: ATP, leucine and tRNA^Leu^ (Boero 2011). The FDA has approved the use of the LeuRS inhibitor tavaborole for the clinical treatment of methicillin‐resistant *S. aureus* (MRSA) and fungal infections, specifically onychomycosis (Pang, Weeks, and Van Aerschot 2021; Rock et al. 2007). MetRS is an enzyme that attaches tRNA to methionine for elongation in protein synthesis and also attaches the initiator tRNA to methionine for protein synthesis (Ojo et al. 2016). The MetRS inhibitor REP8839 showed significant antimicrobial activity against *Streptococcus pyogenes, S. aureus* and vancomycin‐resistant *E. faecalis* (Ochsner et al. 2005; Pang, Weeks, and Van Aerschot 2021).

**FIGURE 1:**
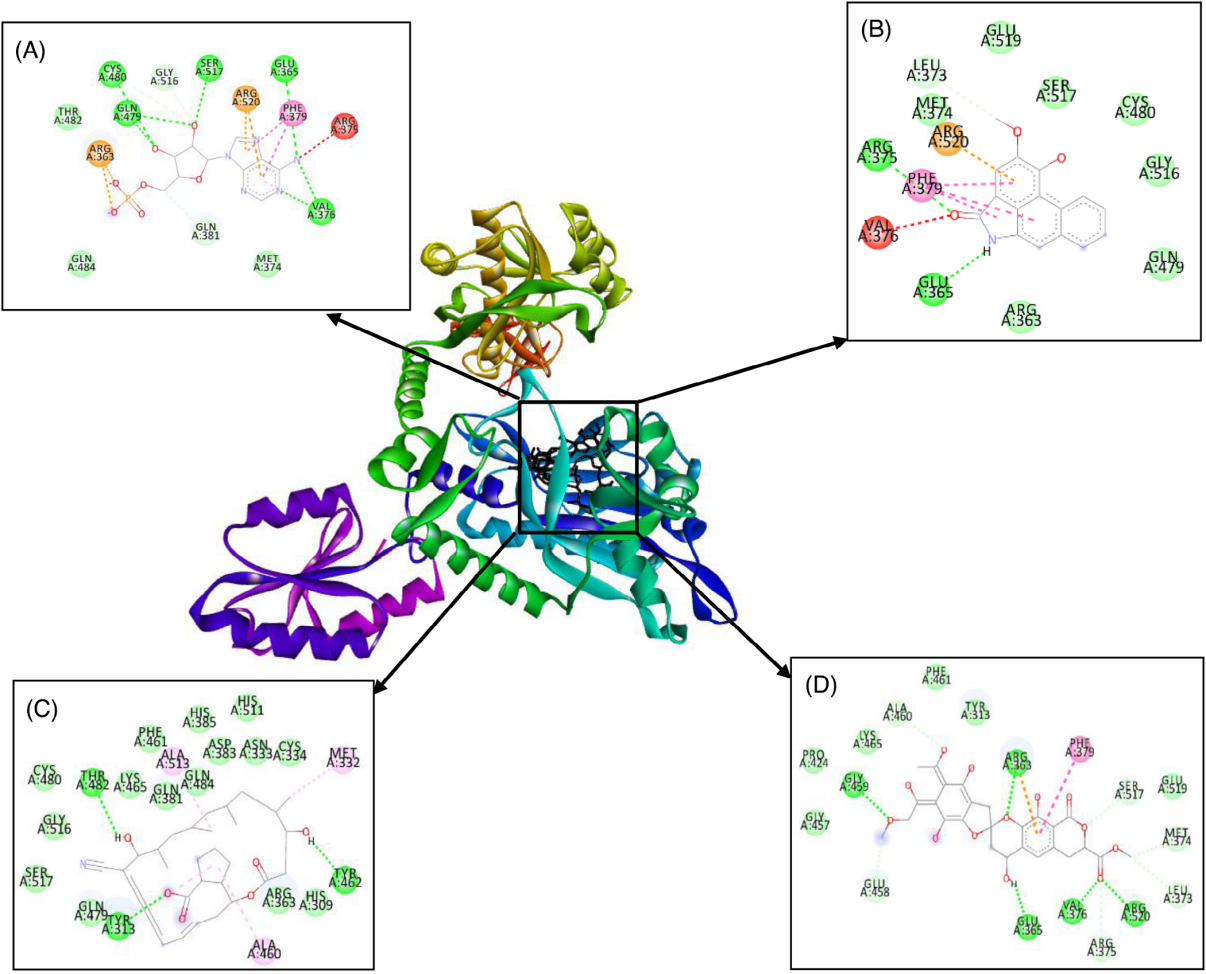
Molecular interaction of (A) AMP (native), (B) piperolactam A, (C) borrelidin and (D) purpuromycin with ThrRS enzyme. ThrRS, threonyl‐tRNA synthetase.

**FIGURE 2:**
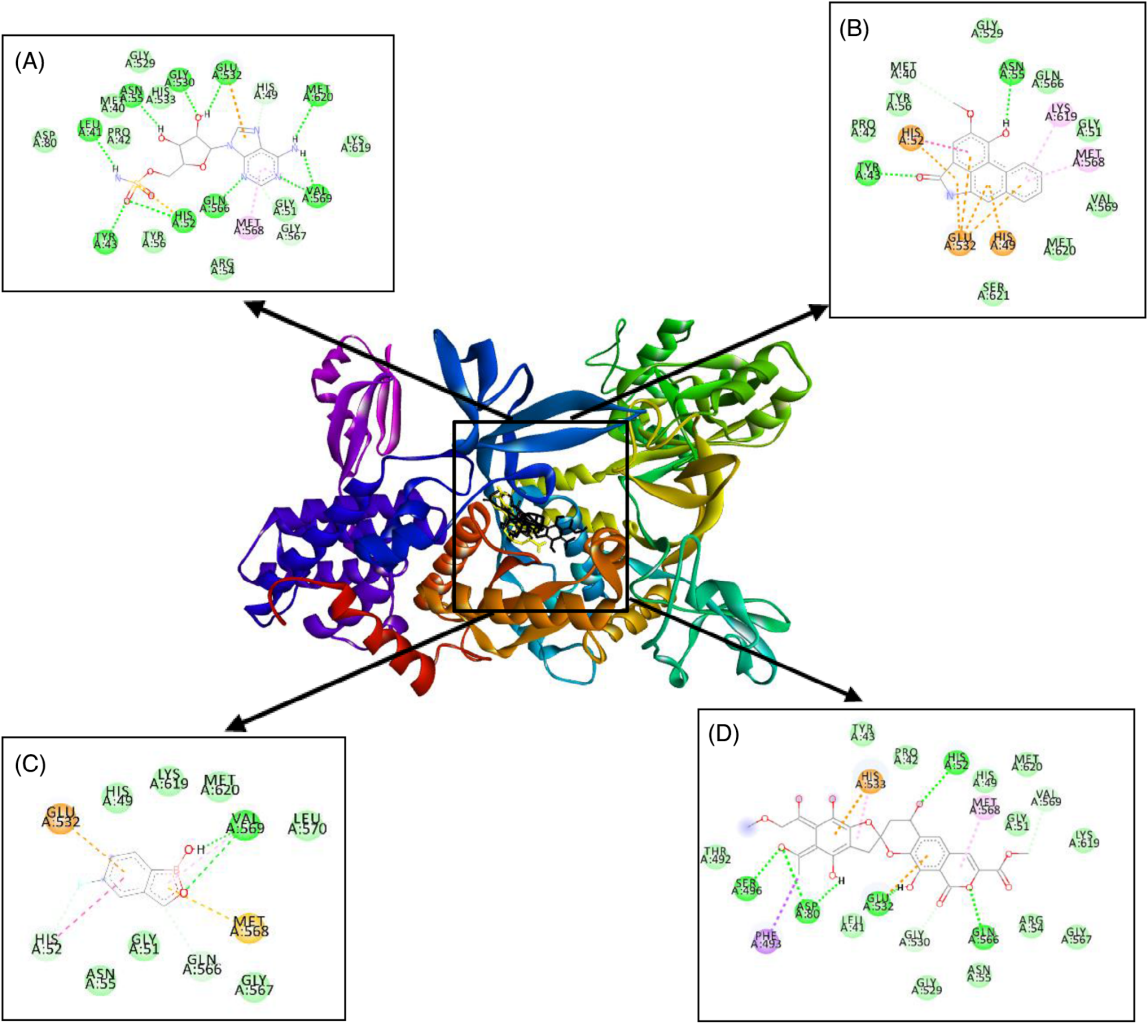
Molecular interaction of (A) LMS (native), (B) piperolactam A, (C) tavaborole and (D) purpuromycin with LeuRS enzyme. LeuRS, leucyl‐tRNA synthetase.

**FIGURE 3:**
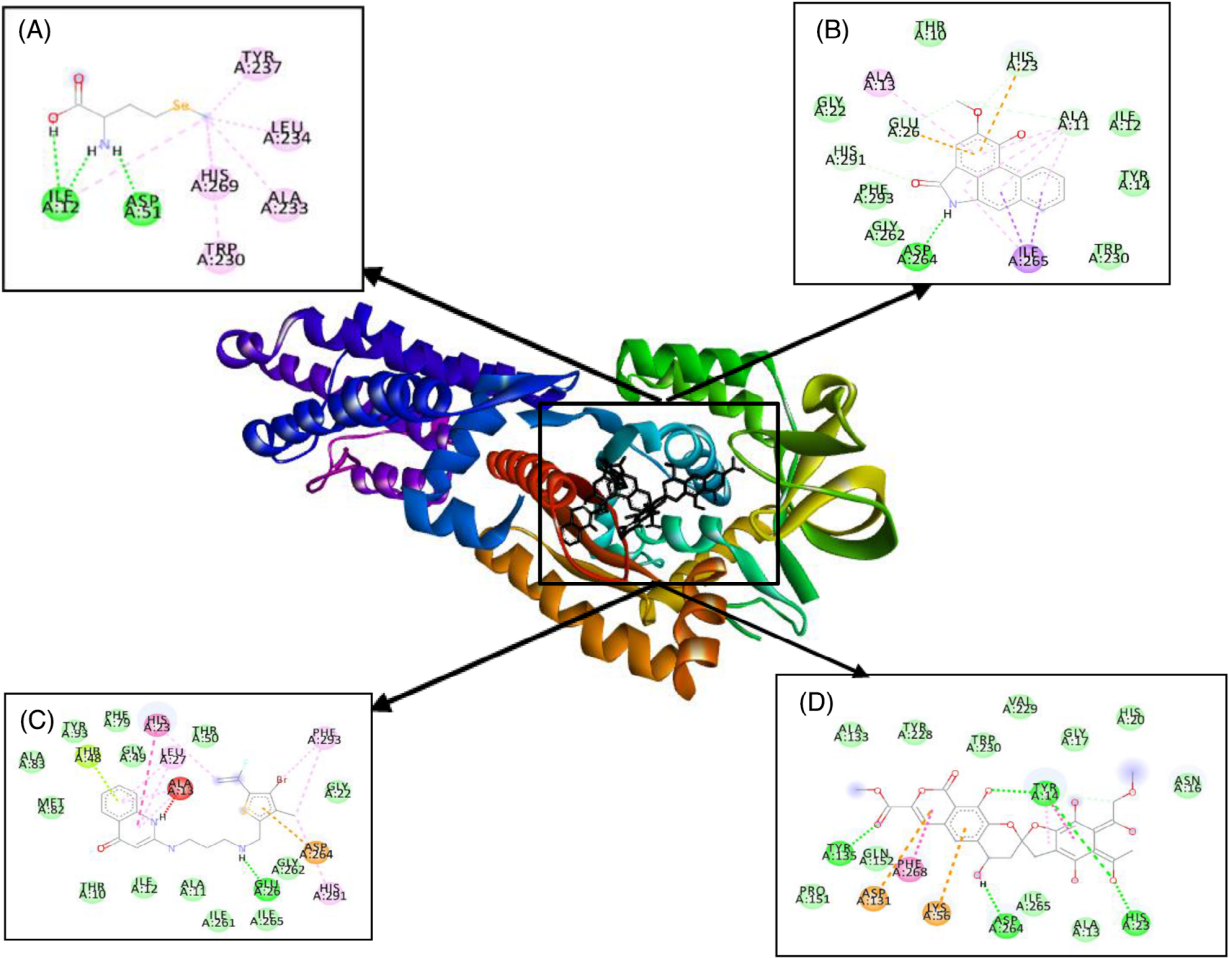
Molecular interaction of (A) MSE (native), (B) piperolactam A, (C) REP8839 and (D) purpuromycin with MetRS enzyme. MetRS, methionyl‐tRNA synthetase.

On the basis of the docking results, the binding affinity value of piperolactam A is slightly lower than that of the native ligand or any other positive control on various protein targets, −7.35 for ThrRS, −7.47 for LeuRS and −6.88 kcal/mol for MetRS, respectively. The binding affinity of purpuromycin was found to be lower than that of any other ligand; purpuromycin will inhibit the acylation of all tRNAs by binding non‐selectively to the tRNA molecule (Cochrane, Norquay, and Vederas 2016). Piperolactam A has potential as an antibacterial agent due to its lower binding affinity value compared to other ligands, although it still has a higher value than purpuromycin. Meanwhile, as shown in Figures 1, 2, 3, the hydrogen bond interactions between receptor and ligand showed that piperolactam A has conventional hydrogen bonds on Glu365, Arg375 for ThrRS, Tyr43 and Asn55 for LeuRS and Asp264 for MetRS. This is related to the absence of hydroxyl in the compound. High‐affinity ligands necessitate strong hydrogen bonds. Additionally, the protein prefers to act as an H‐bonding donor. The ThrRS showed hydrophobic interaction with Phe379 and Arg520 residues. The LeuRS interacted with His49, His52, Glu532, Met568 and Lys619 residues. The MetRS showed hydrophobic interaction on Ala11, Ala13, His23, Glu26 and Ile265 residues. In addition to hydrogen‐bonding and hydrophobic interactions on the aromatic ring, inhibition mechanism studies are also influenced by increased molecular interactions by Van Der Waals (Pantsar and Poso 2018).

On the basis of the data of molecular interaction, piperolactam A and purpuromycin bind to the same active side amino acid residue of ThrRs enzyme Glu365 by forming hydrogen bonds, whereas borrelidin does not bind to this active side residue, as might be seen in Figure 1. In LeuRS enzyme, piperolactam A, tavaborole and puprpuromycin bind to the same active side residues (Glu532 and Met568), although with differences in the type of binding that occurs (Figure 2). However, on binding to the MetRS enzyme, piperolactam A and REP8839 bind to the same active residue sites (Ala13 and His23) (Figure 3). Therefore, on the basis of the binding that occurs between piperolactam A that binds to the active side of the amino acid residues of these enzymes, piperolactam A was predicted to inhibit these three enzymes (ThrRS, LeuRS and MetRS), even though the binding affinity value is not lower than some positive controls.

## 5 Conclusion

Piperolactam A was successfully isolated from *P. betle*. This study utilized in silico docking to predict the binding affinities of compounds with specific aaRSs. These predictions provide valuable insights into potential interactions but do not directly measure or imply antibacterial activity. Further empirical testing is required to evaluate the actual antibacterial efficacy of these compounds in whole‐cell assays.

## Author Contributions

**Diding Latipudin**: conceptualization, data curation, project administration, resources, validation, visualization, writing – original draft and writing – review and editing. **Sefren Geiner Tumilaar**: data curation, formal analysis, investigation, methodology, software, visualization and writing – original draft. **Yoga Ramdani**: data curation, investigation, methodology, resources and software. **Dudi Dudi**: formal analysis, investigation, project administration, supervision, validation and writing – review and editing. **Dikdik Kurnia**: conceptualization, data curation, formal analysis, funding acquisition, project administration, resources, software, supervision, validation and writing – review and editing.

## Ethics Statement

The authors have nothing to report.

## Conflicts of Interest

The authors declare no conflicts of interest.

### Peer Review

The peer review history for this article is available at https://publons.com/publon/10.1002/vms3.70042.

## Data Availability

The authors have nothing to report.
